# Utility of TMPRSS4 as a Prognostic Biomarker and Potential Therapeutic Target in Patients with Gastric Cancer

**DOI:** 10.1007/s11605-021-05101-2

**Published:** 2021-08-11

**Authors:** Hirofumi Tazawa, Takahisa Suzuki, Akihisa Saito, Akira Ishikawa, Toshiaki Komo, Haruki Sada, Norimitsu Shimada, Naoto Hadano, Takashi Onoe, Takeshi Sudo, Yosuke Shimizu, Kazuya Kuraoka, Hirotaka Tashiro

**Affiliations:** 1 Department of Surgery, Kure Medical Center・Chugoku Cancer Center, 737-0023, 3-1, Kure, Hiroshima, Japan; 2 Department of Gastroenterological and Transplant Surgery, Graduate School of Biochemical and Health Sciences, Hiroshima University, Hiroshima, Japan; 3 Department of Diagnostic Pathology, Kure Medical Center・Chugoku Cancer Center, Kure, Hiroshima, Japan

**Keywords:** Gastric cancer, TMPRSS4, Biomarker

## Abstract

**Background:**

Transmembrane serine protease 4 (TMPRSS4) belongs to the family of type II transmembrane serine proteases that are known to be upregulated in many malignant tumors. However, there is a paucity of studies documenting the clinical impact and biological effects of TMPRSS4 on gastric cancer (GC) patients who underwent surgery.

**Methods:**

Tissues samples were obtained from 105 patients with GC who underwent gastrectomy followed by adjuvant chemotherapy, excluding those at stage I. The expression of TMPRSS4 was examined through immunohistochemical analysis. The association between TMPRSS4 expression and clinico-pathological features as well as prognosis was assessed. Moreover, the effects of TMPRSS4 expression on cell migration and sensitivity to 5-FU were investigated.

**Results:**

The expression rate of TMPRSS4 was 56.3% (59/105) in GC cases. The expression of TMPRSS4 was positively correlated with the depth of tumor (T) and venous (V) invasion. The 5-year overall survival (OS) and recurrence-free survival (RFS) rates of the TMPRSS4-positive group was significantly lower than that of the TMPRSS4-negative group (*p*=0.0001 and *p*=0.005, respectively). Especially, there was significant differences in OS and RFS of patients with stage III cancer between the two groups (*p*=0.0064 and 0.012, respectively). Multivariate analysis demonstrated that TMPRSS4 expression and the stage of cancer were crucial prognostic factors for RFS. TMPRSS4-silenced GC cells exhibited increased sensitivity to 5-FU when compared with the non-specific control siRNA-transfected cells.

**Conclusion:**

TMPRSS4 can be considered as a potential prognostic biomarker, especially for stage III, and a promising therapeutic target for GC.

**Supplementary Information:**

The online version contains supplementary material available at 10.1007/s11605-021-05101-2.

## Introduction

Surgery is considered as the main treatment method for operable gastric cancer (GC). However, appreciable patients at stages II and III undergo recurrence. Therefore, various adjuvant chemotherapy methods have been developed and tested to prevent postoperative recurrence.^1^ In 2007, the Adjuvant Chemotherapy Trial of S-1 for GC (ACTS-GC) demonstrated that surgery in addition to a 12 months of postoperative adjuvant chemotherapy with S-1 improved the overall survival (OS) and recurrence-free survival (RFS) rates compared to surgery alone for patients at stages II and III of GC.^2, 3^ However, the 5-year OS rate in patients at stages IIIA and IIIB were 67.1 and 50.2% in the S-1 group, respectively, indicating the need for further improvement. Therefore, in 2019, through additional therapy of docetaxel to the S-1 group, Japan Clinical Cancer Research Organization (JACRO) GC-07 improved the prognosis of gastric patients at stage III: the 3-year RFS rate in patients at stage III was 66% in the S-1 plus docetaxel group and 50% in the S-1 alone group.^4^ However, the data related to OS was not presented.

Serine proteases are involved in the degradation of basement membrane and extracellular matrix, which allows tumor cells to invade the surrounding tissue. Proteases are involved in the proliferation, migration, invasion, and angiogenesis of cancer cells. Type II transmembrane serine proteases (TTSPs) are a novel subfamily of serine protease and are known to be upregulated in malignant tumors.^5, 6^ Transmembrane serine protease 4 (TMPRSS4) belongs to a family of TTSPs. It is overexpressed in many cancer types, including pancreatic cancer, colon cancer, lung cancer, ovary cancer, hepatocellular carcinoma, and GC.^7–12^ The expression of TMPRSS4 is associated with the epithelial to mesenchymal transition (EMT) and upregulates integrin-α5 to induce invasiveness.^13, 14^ Additionally, TMPRSS4 regulates urokinase-type plasminogen activator (uPA) via a dual mechanism through increasing the gene expression and processing of pro-uPA into its active form, which leads to an enhanced invasion.^15^

Recently, the overexpression of TMPRSS4 has been shown to be associated with poor prognosis in GC patients at stages II and III post-surgery.^16^ However, a few studies have documented the impact of TMPRSS4 on adjuvant chemotherapy in GC patients who underwent surgery. The effect of TMPRSS4 on the sensitivity of GC cells to chemotherapy was not examined yet.

In the present study, we examined the expression of TMPRSS4 in GC tissues and its correlation with clinico-pathological parameters and prognosis in GC patients undergoing adjuvant chemotherapy following surgery. We also investigated the effect of TMPRSS4 expression on the sensitivity to chemotherapy in GC cells.

## Materials and Methods

### Patients

Between January 2012 and January 2015, 105 patients underwent resection for GC at the Department of Surgery, Kure Medical Center and Chugoku Cancer Center, National Hospital Organization. All patients received R0 resection with more than D1 plus or D2 lymph node dissection according to the previously outlined guideline.^17^ Patients with histologically confirmed stage I GC did not receive adjuvant chemotherapy. However, patients with histologically confirmed stage II or III GC received adjuvant chemotherapy consisting of S-1 (80 to 120 mg per day) for 4 weeks, followed by 2 weeks of rest. This 6-week cycle was repeated for 1 year, according to the previously outlined guidelines.^2^ Patients with histologically confirmed stage IV GC received adjuvant chemotherapy consisting of S-1 in addition to cisplatin. No patients received chemotherapy before the operation.

The patient’s medical records were reviewed retrospectively, and data were collected. Following this step, OS and RFS were analyzed. Tumors were classified based on the Japanese Classification of Gastric Carcinoma, 14^th^ edition.^18^ This retrospective study (approval number: 2019-93) was reviewed and approved by the Institution Review Board of Kure Medical Center.

### Immunohistochemical Analysis

Immunohistochemical analysis was carried out following the manufacturer’s instructions (DAKO). Four-micrometer-thick tissue sections were incubated with the primary rabbit anti-TMPRSS4 antibody (1:200; abl50595, Abcam, Cambridge, UK) overnight at 4 °C followed by the incubation with the secondary antibody. All immunohistochemistry results were determined by two independent pathologists (A.S., K.K.) blinded to this study. The percentage of positively stained tumor cells was scored as follows: TMPRSS4-postive, 50% positive and more than 50% positive cells; TMPRSS4-negative, less than 50% positive cells.

### Cell Culture and Reagents

The two human GC cell lines used in this study are as follows: NUGC3 (from JCRB Cell Bank), and MKN45 (from RIKEN Cell Bank). All cell lines were cultured in RPMI-1640 medium (Nacalai Tesque, Kyoto, Japan) supplemented with 10% fetal bovine serum (Life Technologies, Carlsbad, CA) at 37 °C in a humidified atmosphere supplied with 5% CO_2_.

### RNA Interference

#### siRNA Transfection

Cells were transfected with TMPRSS4-siRNA MISSION-siRNA (#SASI_Hs02_00317341, #SASI_Hs02_00317342, and #SASI_Hs02_00317343; Sigma-Aldrich, St. Louis, MO) using the Lipofectamine RNAiMAX reagent (Invitrogen; Thermo Fisher Scientific, Waltham, MA), according to the manufacturer’s instructions. The control siRNA MISSON-siRNA Universal Negative Control (Sigma-Aldrich) was used as a negative control.

### Quantitative Real-Time PCR (qPCR)

Total RNA was extracted using NucleoSpin RNA (Takara Bio, Shiga, Japan) and then was reverse transcribed into cDNA using PrimerScript RT MasterMix (Takara Bio) according to the manufacturer’s protocol. The resulting cDNA was amplified using TB Green Premix Ex Taq II (Takara Bio). The expression of the target gene was quantified using the comparative cycle threshold method. The primer sequences used were as follows: TMPRSS4 forward, 5′-ACTACTGAGCCTGGCGAGTA-3′, and reverse, 5′-TTGACACAGTGCTCCTCGTC-3′, and GAPDH forward, 5′-CAACGGATTTGGTCGTATTGG-3′, and reverse, 5′-CCATGGGTGGAATCATATTGG-3′.

### Western Blotting

Cells were washed with PBS and lysed with Cell Lysis Buffer (Cell Signaling, Beverly, MA) containing PMSF (Cell Signaling). Equal amounts of sample proteins were denatured through boiling for 10 min in SDS-sample buffer containing 1% beta-mercaptoethanol. The proteins were then electrophoresed on an SDS–polyacrylamide gel and transferred onto a PVDF membrane (Bio-Rad, Hercules, CA). The membrane was blocked using 5% skim milk prepared in Tris-buffered saline containing 0.05% Tween 20 for 1 h, and thereafter, it was incubated with a primary antibody overnight. The primary antibodies used are as follows: anti-TMPRSS4 rabbit polyclonal antibody (1:2000;11283-1, Proteintech) and anti-beta actin mouse monoclonal antibody (1:10,000; A5441, Sigma-Aldrich). Proteins were then visualized with an anti-mouse and rabbit IgG horseradish peroxidase-conjugated secondary antibody (1:3,000; Cell Signaling) using the ECL Western Blotting Detection System (Bio-Rad Technologies).

### Wound-Healing Assay

Cells were transfected with the above-indicated siRNAs for 72 h. Cell monolayers were scratched and then incubated in medium with 10% FBS. About 24/48 h after scratching, the percent reduction of the scratched area was evaluated using BZ-X700 (Keyence, Osaka, Japan).

### Proliferation Assay

Cells were seeded at a concentration of 4,500 cells (NUGC3), and 3,000 cells (MKN45) per well in a 96-well plate and transfected with the above-indicated siRNAs for 72 h. After transfection, the cells were exposed to different concentrations of 5-fluorouracil (5-FU) for 72 h. Thereafter, cell proliferation was evaluated using the Cell Counting Kit-8 (CCK-8, Dojindo, Kumamoto, Japan). The optical absorbance of samples was measured using the fluorescence microplate reader MULTISKAN GO (Thermo Fisher Scientific, Fremont, CA) at a wavelength of 450 nm and applied to the following formula: Relative cell viability = Number of cells treated with drugs/Number of cells treated with vehicle. 5-FU was purchased from Towa pharmaceutical (Osaka, Japan).

### Statistical Analysis

The continuous variables were expressed as median (range) and were analyzed using Mann-Whitney *U*-test. The categorical variables and postoperative courses were compared using *χ*^2^ tests with Yates correction. OS and RFS after surgical resection were compared in each group using Kaplan-Meier method and log-rank test. Univariate and multivariate analyses were performed using Cox regression test. The difference was considered statistically significant if the *p* value was less than 0.05. Statistical analyses were performed using the SPSS statistical software version 16 (Chicago, Illinois, USA).

## Results

### Overexpression of TMPRSS4 in GC Tissues

**Fig. 1:**
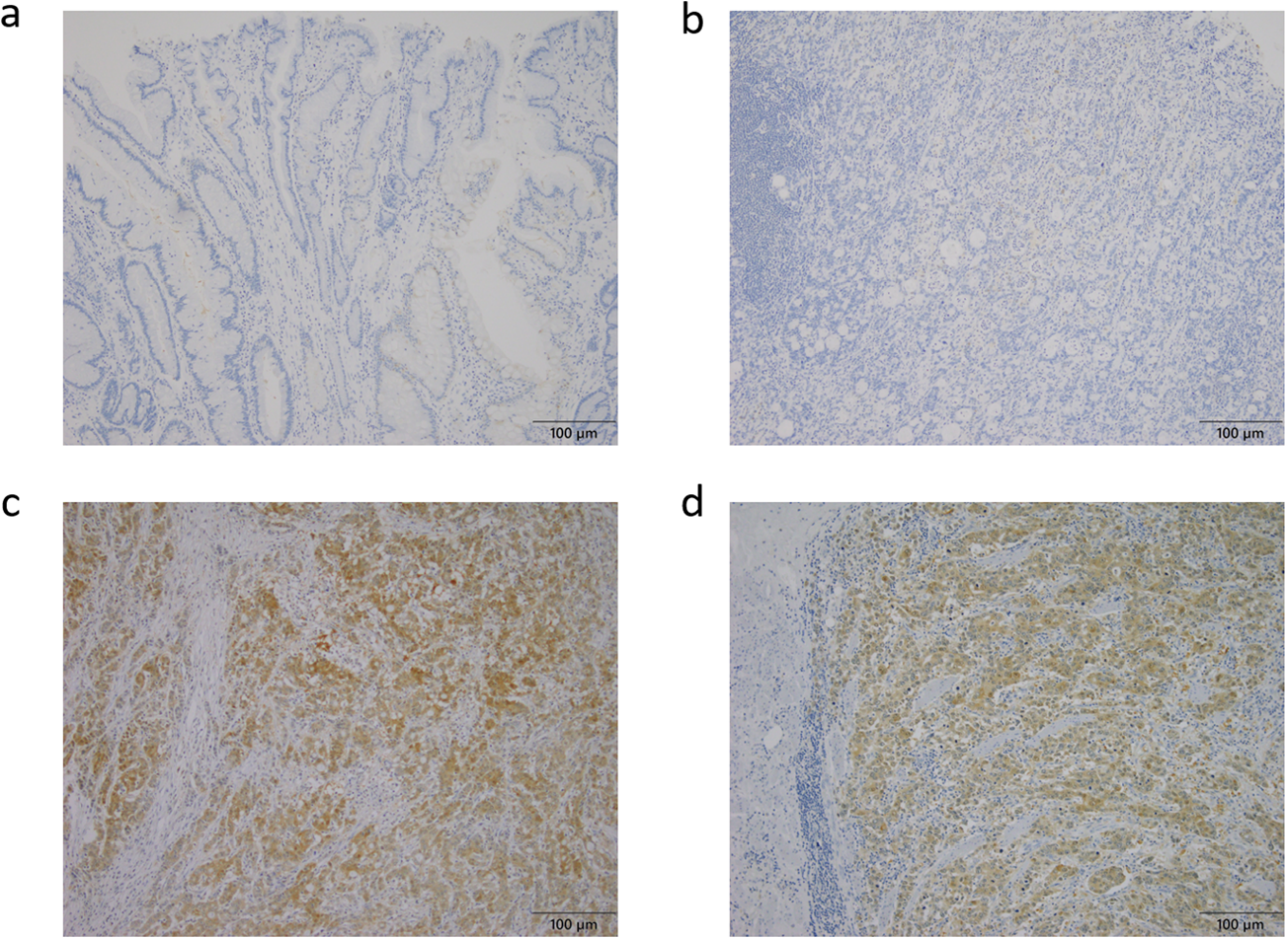
Immunohistochemical staining of TMPRSS4 in GC lesions and non-cancerous tissues. **a** TMPRSS4-negative non-cancerous gastric tissues. **b** TMPRSS4-negative GC tissues with moderately differentiated adenocarcinoma. **c** TMPRSS4-positive GC tissues with moderately differentiated adenocarcinoma. **d** TMPRSS4-positive GC tissues with poorly differentiated adenocarcinoma (original magnification, 40×). Scale bar indicates 100 μm.

TMPRSS4-positive staining was mainly observed in the cytoplasm of GC cell using immunohistochemical analysis (Fig. 1). The expression rate of TMPRSS4 was 56.3% (59/105) in all GC cases (Table 1).

**Table 1:** Correlation between TMPRSS4 and clinical features

Variables	TMPRSS4 expression		*p* value
	Negativity	Positivity	
	*N*=46	*N*=59	
Age	66.7 (38–87)	71.5 (45–91)	0.2396
Gender (male/female)	32/14	40/19	0.9302
Tumor location (J/U/M/L)	2/4/20/18	5/11/20/23	0.0996
Tumor size	53.3 (7-200)	62.1 (15–167)	0.6574
pStaging (I/II/III/IV)	20/10/14/2	11/16/24/8	0.1876
Depth of tumor invasion (T) (1/2/3/4)	18/6/16/6	8/5/34/12	0.0258
Lymph node metastasis (N) (0/1/2/3)	19/7/8/12	17/12/10/20	0.6475
Lymphatic invasion (ly) (0/1/2/3)	24/10/6/6	18/14/11/15	0.2152
Venous invasion (v) (0/1/2/3)	34/9/3/0	26/22/8/3	0.0108
Histology (tub/por/other)	20/22/4	18/35/6	0.6868
Tumor infiltrative pattern (IFN) (a/b/c)	14/14/9	13/19/19	0.1094
Lauren classification (i/m/d)	13/17/8	18/21/12	0.2033

*J* esophagogastric junction, *U* upper third, *M* middle third, *L* lower third, *i* intestinal type, *m* mixed type, *d* diffuse type

### Association Between Clinico-pathological Features and TMPRSS4 Positivity

We evaluated the association of TMPRSS4 positivity with clinico-pathological features (Table 1). Regarding the tumor location, the TMPRSS4-positive cells were higher in the proximal site than the distal site of stomach without any significance. The expression of TMPRSS4 was positively and significantly correlated with the depth of tumor invasion (T) and venous invasion (v), but not with the age, gender, tumor size, lymph node metastasis (N), histological type, and stages of the tumor.

### Association Between the Expression of TMPRSS4 and the Outcomes of Patients After Gastrectomy

**Fig. 2:**
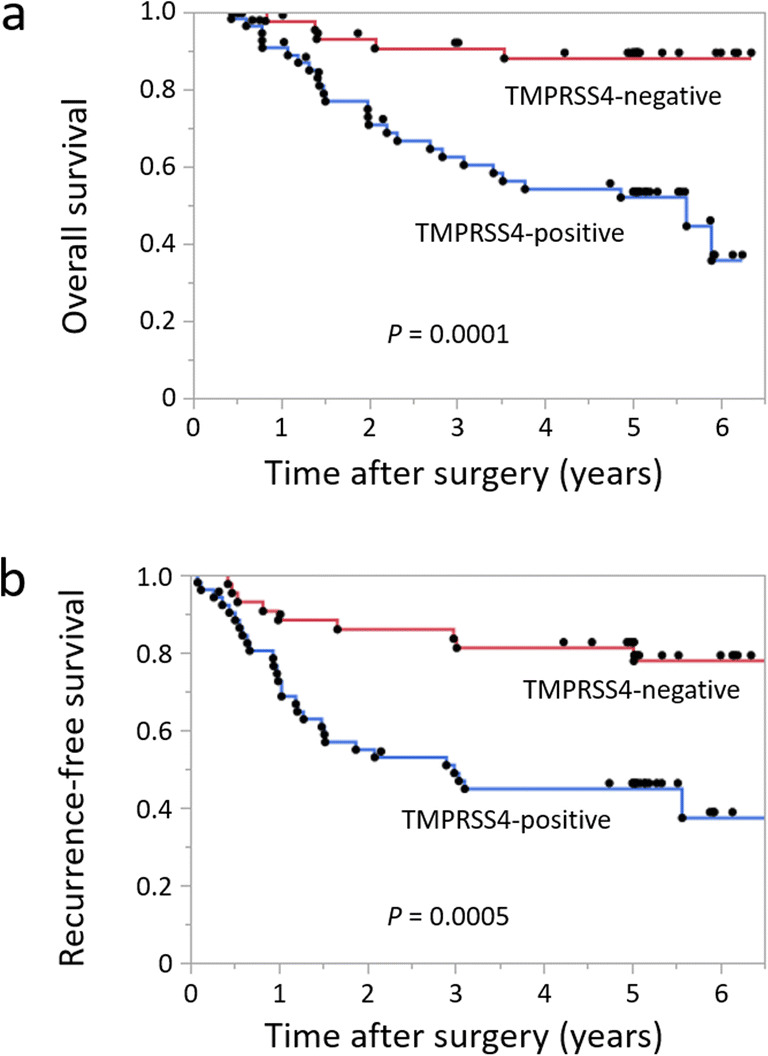
Kaplan-Meier curves for all GC patients classified based on the expression of TMPRSS4. **a** Overall survival (OS) curves of all GC patients. **b** Recurrence-free survival (RFS) curves of all GC patients. The OS and RFS outcomes were compared using the log-rank test.

The 5-year OS rate of the TMPRSS4-positive group was significantly lower than that of the TNMPRSS4-negative group (51% vs 87%, *p*=0.0001). Moreover, the 5-year RFS rate was also significantly lower in the TMPRSS4-positive group compared with the TMPRSS4-negative group (43% vs 83%, *p*=0.005) (Fig. 2a, b). Next, the univariate and multivariate analyses were conducted using Cox proportional hazard model to evaluate the influence of TMPRSS4 expression and pathological factors on the prognosis of GC patients who underwent gastrectomy. In regard to OS, the univariate analysis revealed that TMPRSS4, tumor size, deeper tumor invasion, lymph node metastasis, venous invasion, lymphatic invasion, invasion type, and stages of tumor were all significant prognostic factors for OS. However, the multivariate analysis revealed that the stages of tumor and venous invasion were significant prognostic factors for OS, but TMPRSS4 was not a significant prognostic factor for OS (Table 2). In regard to RFS, the univariate analysis revealed that the gender, Lauren’s pathological type, TMPRSS4, tumor size, deeper tumor invasion, lymph node metastasis, venous invasion, lymphatic invasion, invasion type, and stages of tumor were all significant prognostic factors for RFS. Moreover, the multivariate analysis revealed that TMPRSS4 and the stages of tumor were also significant prognostic factors for RFS (Table 3).

**Table 2:** Univariate analyses of OS and RFS in patients with gastric cancer

Variables	Number of patients	OS		RFS	
		5-year survival rate (%)	*p* value	5-year survival rate (%)	*p* value
Age			0.713		0.2158
70 <		68.9		65.8	
70 ≥		65.8		57.0	
Gender			0.4		0.0229
Male	72	64.6		53.0	
Female	33	73.4		82.1	
TMPRSS4			0.0001		0.0005
Positivity	59	52.0		44.9	
Negativity	46	88.0		81.2	
Tumor location			0.106		0.1842
Junction	7	71.4		71.4	
Upper	17	43.8		40.0	
Middle	40	77.3		71.0	
Lower	41	68.1		59.1	
Tumor size			0.0019		0.0334
52 <		53.4		73.5	
52 ≥		83.9		47.8	
pStage			0.0001		0.0001
I	31	100.0		90.3	
II	25	79.0		63.3	
II	39	49.1		36.4	
IV	10	10.0			
Depth of tumor invasion(T)			0.0001		0.0003
T1	28	96.3		89.3	
T2	11	77.8		70.0	
T3	49	69.1		51.1	
T4	17	11.7		20.0	
Lymph node metastasis (N)			0.0001		0.0001
0	38	96.7		78.6	
1	18	88.2		88.2	
2	20	49.9		35.1	
3	29	30.2		30.2	
Lymphatic invasion (ly)			0.0168		0.0342
0	44	79.9		74.7	
1	24	68.7		59.9	
2	16	67.5		50.0	
3	21	39.7		41.3	
Venous invasion (v)			0.0058		0.0027
0	61	79.9		77.9	
1	30	51.4		39.3	
2	11	33.8		33.8	
3	3	66.7		50.0	
Histology			0.4996		0.6319
tub	40	75.2		67.3	
por	55	61.8		57.0	
Other	10	66.7		60.0	
Tumor infiltrative type (IFN)			0.0007		0.0026
a	28	79.6		63.0	
b	35	74.9		62.6	
c	30	34.7		36.1	
Lauren classification			0.1586		0.042
i	40	61.9		52.8	
m	32	76.0		56.7	
d	21	46.4		61.4	

*OS* overall survival, *RFS* recurrence-free survival

**Table 3:** Multivariate analyses of OS and RFS in patients with gastric cancer

		OS			RFS	
	HR	95% CI	*p* value	HR	95% CI	*p* value
Venous invasion	2.79	1.150-3.740	0.0016			
pStage	1.94	0.016-2.922	0.012	2.03	1.295–6.892	0.0094
TMPRSS4				2.89	−1.3314 to −0.2897	0.0013

*OS* overall survival, *RFS* recurrence-free survival, *HR* hazard ratio, *CI* confidence interval

**Fig. 3:**
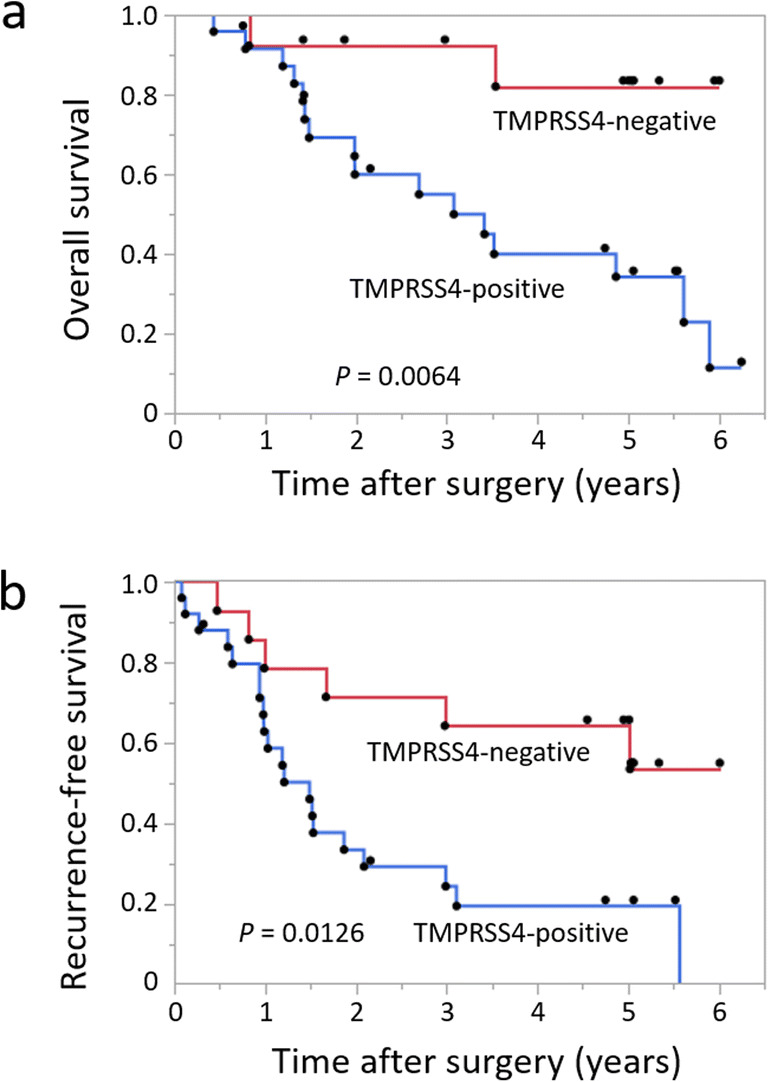
Kaplan-Meier curves for all GC patients classified based on the expression of TMPRSS4 in stage III. **a** OS curves of GC patients in stage III. **b** RFS curves of GC patients in stage III. The OS and RFS were compared using the log-rank test.

In terms of clinical stages, there was significant differences in OS and RFS of patients with stage III GC between the two groups (*p*=0.0064 and *p*=0.012, respectively): the 5-year OS rates of TMPRSS4-positive and TMPRSS4-negative groups were 38% and 85%, respectively, and the 5-year RFS rates of TMPRSS4-positive and TMPRSS4-negative group were 18% and 67%, respectively (Fig. 3a, b). For the patients at stage I, the 5-year OS rates in both groups were 100% and 100%, respectively, and there was no significant difference in the RFS rates between the two groups, although TMPRSS4-positive group exhibited lower RFS rate than TMPRSS4-negative group (Fig. S1a, b). Also, there was no significant difference in OS and RFS rates of patients with stages II GC. (Fig. S1c, d). In regard to stage IV, the number of patients at stage IV was 10, among which there was only 2 TMPRSS4-negative cases, which was too small to analyze the OS.

### Silencing of TMPRSS4 Reduces the Migration of GC Cells and Enhances the Sensitivity to 5-FU

**Fig. 4:**
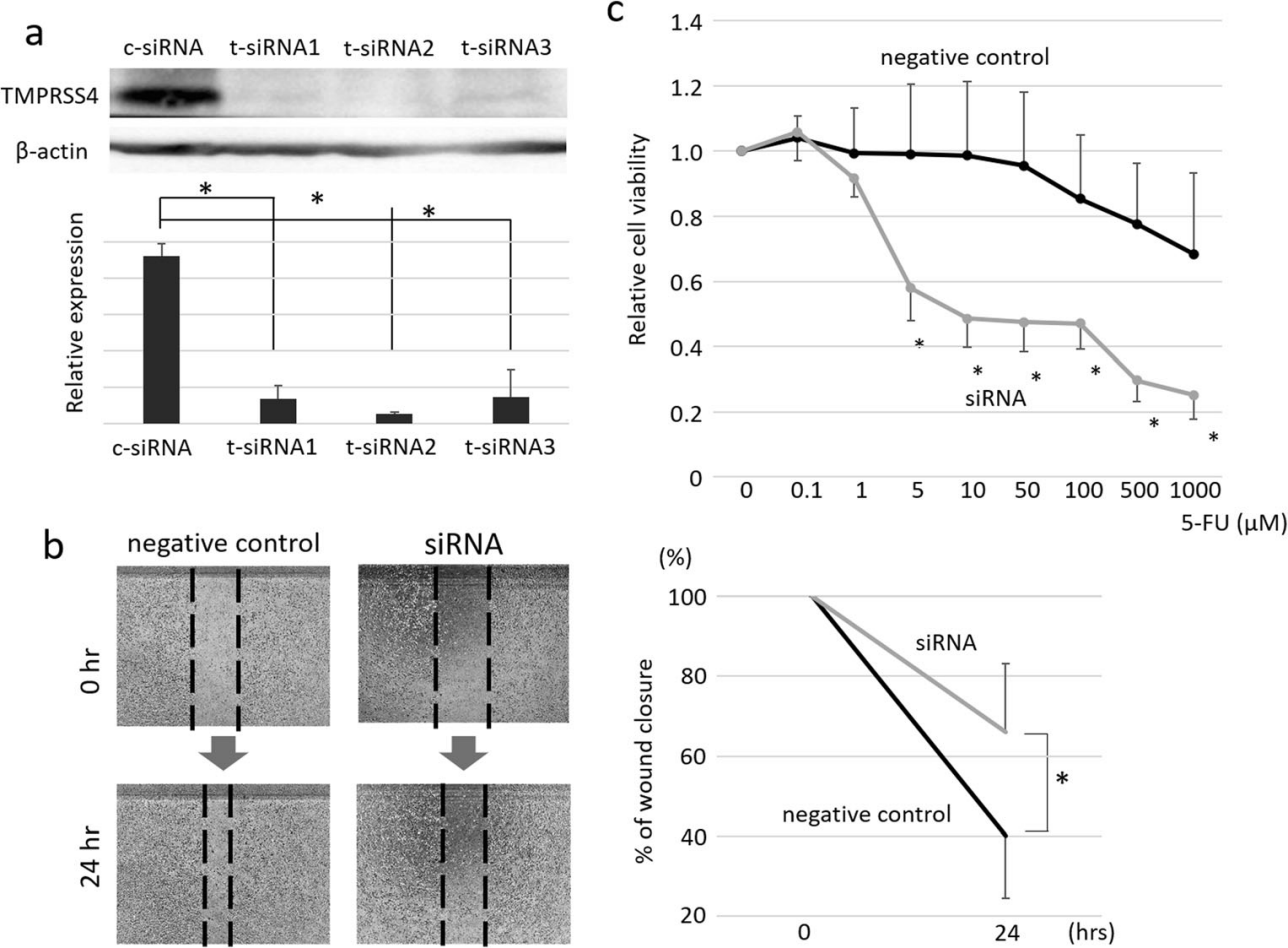
TMPRSS4-silencing reduces the migration and chemosensitivity to 5-FU in GC cells. **a** mRNA levels of TMPRSS4 in NUGC-3 cells transfected with control siRNA (c-siRNA) or TMPRSS4-targeting siRNA (t-siRNA). Control siRNA transfected cells were used as control. Western blot analysis of TMPRSS4 protein levels in NUGC-3 cells transfected with control siRNA (c-siRNA) or TMPRSS4-targeting siRNA (t-siRNA). Control siRNA transfected cells were used as control. Bar graphs indicate densitometric analysis of western blots from three independent experiments. (1, negative control; 2, TMPRSS4-targeting siRNA #1; 3, TMPRSS4-targeting siRNA #2; and 4, TMPRSS4-targeting siRNA #3). **b** Representative images of the migrated cells taken at 24 h after scratching. Bar graphs indicate the mean percentage (± SEM) of wound closure. **c** Proliferation assay using CCK-8 kit were performed to examine the inhibitory effect of 5-FU. ^*^p<0.05

Western blot analysis revealed that TMPRSS4 protein was differentially expressed in both NUGC-3 and MKN-45 cells (Fig. 4a, S2a). The delivery of TMPRSS4-targeting siRNA led to a significant decrease in mRNA and protein levels of TMPRSS4 in both MKN-45 and NUGC-3 cells (Fig. 4a, S2a). Cell proliferation assay revealed that TMPRSS4-silencing did not reduce the proliferation of the two GC cell lines. However, the wound-healing assay revealed that TMPRSS4-silencing significantly suppressed the wound healing when compared to the control siRNA-transfected cells (*p*=0.03) (Fig. 4b, S2b).

Thereafter, we tested the effect of 5-FU on GC cells. TMPRSS4-silenced NUGC-3 and MKN-45 cells were found to be highly and significantly sensitive to 5-FU compared to the corresponding non-specific control siRNA-transfected cells (Fig. 4c, S2c).

## Discussion

In the present study, we evaluated whether the expression of TMPRSS4 is associated with the clinico-pathological factors and the outcomes after gastrectomy for the patients with GC. Additionally, we examined the effect of TMPRSS4 expression in vitro on the proliferation, migration, and the sensitivity to chemotherapy of GC cells. Our results showed that the expression of TMPRSS4 is significantly correlated with deeper tumor and venous invasion. Moreover, GC patients with positive expression of TMPRSS4 exhibited poor RFS. Especially, in the subgroup of patients with stage III GC, the positive expression of TMPRSS4 was significantly correlated with poor OS and RFS after gastrectomy, as compared to the negative expression of TMPRSS4. Low expression of TMPRSS4 suppressed the migration of GC cells, but not the proliferation. However, low expression of TMPRSS4 in GC cells enhanced their sensitivity to 5-FU. These results suggest that TMPRSS4 is a potential prognostic factor for the recurrence of GC and, thus, can be considered as a promising therapeutic target.

Several studies have been conducted to identify the useful biomarkers for predicting the outcomes of GC patients at stages II and III.^19–25^ Kim MH. et al. focused on thymidylate synthetase (TS), which influences the response to fluoropyrimidine-based chemotherapy, including TS-1 and capecitabine.^24^ In classical trial, they showed that high TMPRSS4 expression is a predictive factor for the worse outcomes during capecitabine plus oxaliplatin adjuvant chemotherapy.^26, 27^ However, the number of patients with high TMPRSS4 expression was small (15.8%), and there was no significant difference in the outcomes between stages II and III patients; however, stage III patients did show shorter OS and DFS than those at stage II.^24^ There were several reports regarding programmed cell death ligand-1 (PD-L1) in GC.^24, 25^ However, there were no association between the expression of PD-L1 and the outcomes in adjuvant chemotherapy subgroup of stages II and III patients. Kikuchi et al. using proteome analyses showed that the overexpression of ephrin A2 receptors in cancer stromal cells act as a prognostic factor for the relapse of GC in patients with S-1 adjuvant chemotherapy.^21^ This biomarker could be promising; however, there was no significant difference noted in OS, and the authors stated that the number of patients was too small to indicate a statistical difference. Our multivariate analysis showed that TMPRSS4 was a significant prognostic factor for the recurrence in all GC patients who underwent curative operation. This result indicates that TMPRSS4 can be applied as a useful biomarker for the recurrence in patients undergoing curative operation. We think that TMPRSS4 exhibits the advantage of high expression rate, because the number of patients with high TMPRSS4 expression (56.3%) was high in our case. However, our study is single institutional, and the number of patients is also small. Therefore, a large-scale study is needed to validate the usefulness of TMPRSS4 as a biomarker for successful prognosis of GC. Our results are in accordance with the previous report published by Sheng. et al.^16^ They showed that the 5-year survival rates of TMPRSS4-positive and negative patients with stages II and III GC were approximately 40% and 80%, respectively, further proposing that TMPRSS4 expression can be applied as an indicator of poor prognosis in GC patients. However, this study did not include the adjuvant chemotherapy. In our study, all GC patients at stages II and III received adjuvant S-1 monotherapy.

Our results showed that the 5-year OS and RFS rates of TMPRSS4-negative patients with stage III GC were significantly higher than those of TMPRSS4-positive patients with stage III GC. The downregulation of TMPRSS4 induced an increased susceptibility to 5-FU. These results suggest the possibility that TMPRSS4-negative expression increases chemo-sensitivity, resulting in the suppression of recurrence in TMPRSS4-negative GC patients at stage III. ACTS-GC trial demonstrated that the 5-year OS rate in patients with stage IIIB GC undergoing S-1 adjuvant chemotherapy was 50.2%, suggesting that it can be improved further.^3^ Therefore, the addition of docetaxel to S-1 adjuvant chemotherapy, in JACCROGC-07 trial, improved the 3-year RFS rate in patients with stage III GC, compared with S-1 alone, although the data related to OS was not presented.^4^ In Japan, S-1 plus docetaxel replaced S-1 monotherapy in patients with stage III GC since it was published. However, S-1 plus docetaxel therapy likely induces grade 3 or greater adverse events, such as neutropenia and alopecia, compared with S-1 alone adjuvant therapy. Therefore, the expression of TMPRSS4 may be a useful predictor for modulating the S-1 plus docetaxel therapy in patients with stage III GC, because the prognosis of TMPRSS4- negative patients at stage III was notable under only S-1 monotherapy, as the 5-year survival rate was 85%.

In the present study, we showed that low expression of TMPRSS4 suppressed the migration of GC cells and enhanced the sensitivity to 5-FU. These results were in accordance with the previous reports.^9, 28^ Exposito et al. showed using lung cancer cell lines that the downregulation of TMPRSS4 could sensitize cells to the cytotoxic effect of several chemotherapy drugs, including 5-FU. The knockdown of TMPRSS4 in lung cancer cells induced the downregulation of thymidylate synthetase (TS). TS catalyzes the conversion of dUMP to dTMP, which is required for DNA synthesis and repair. Basically, high TS expression has been shown to result in relatively low sensitivity to fluoropyrimidine-based chemotherapy, including S-1.^29, 30^ Downregulation of TMPRSS4 may sensitize cancer cells to the cytotoxic effects of S-1 via decreased expression of TS. Conversely, Sasako et al. showed a significant association between high TS expression and an enhanced outcome in ACTS-GC.^20^ Therefore, it is required to conduct further studied to clarify this discrepancy.

In the subgroup of patients with stage II GC, there was no significant difference in the OS between the positive and negative expression of TMPRSS4, although the OS of GC patients with negative expression of TMPRSS4 was higher. The number of patients in the subgroup of stage II GC was small. Therefore, it is required to conduct a study with large sample size to validate the impact of TMPRSS4 on stage II GC.

TMPRSS4 is considered as a novel potential therapeutic target in GC.^31^ Our study showed that low expression of TMPRSS4 suppressed the migration of GC cells and enhanced the sensitivity to 5-FU. These results suggest that TMPRSS4 may be considered as a good target candidate. The pharmacological compounds to inhibit TMPRSS4 in combination with chemotherapy, such as S-1, may promote the sensitivity to chemotherapy through inhibiting the function of TMPRSS4. Recently, 2-hydroxydiarylamide derivatives, IMD-0354 and KRT1853, were developed as TMPRSS4 serine protease inhibitors.^32^ These new compounds have been shown to inhibit cancer cell invasion, migration, and proliferation; however, the sensitivity to chemotherapy was not determined.

In conclusion, TMPRSS4 can be considered as a potential prognostic biomarker for GC, especially in stage III, and a promising therapeutic target.

## Supplementary Information

FIG. S1 Kaplan-Meier curves for all GC patients classified based on the expression of TMPRSS4 in stages I and II. **a.** OS curves for all GC patients in stage I. **b.** RFS curves for all GC patients in stage I. **c.** OS curves for all GC patients in stage II. **d.** RFS curves for all GC patients in stage II. The OS and RFS were compared using the Log-rank test. (PNG 540 kb)

High Resolution image (TIF 253 kb)

FIG. S2 TMPRSS4-silencing reduces the migration and chemosensitivity to 5-FU in GC cells. **a.** mRNA levels of TMPRSS4 in MKN-45 cells transfected with control siRNA (c-siRNA) or TMPRSS4-targeting siRNA (t-siRNA). Control siRNA transfected cells were used as control. Western blot analysis of TMPRSS4 protein levels in MKN-45 cells transfected with control siRNA (c-siRNA) or TMPRSS4-targeting siRNA (t-siRNA). Control siRNA transfected cells were used as control. Bar graphs indicate the densitometric analysis of western blots of three independent experiments. **b.** Representative images of migrated cells taken at 48 h after scratching. Bar graphs indicate the mean percentage (± SEM) of wound closure. **c.** Proliferation assay using CCK-8 kit was performed to examine the inhibitory effect of 5-FU. ^*^p<0.05 (PNG 1216 kb)

High Resolution image (TIF 528 kb)
