# Supplementary figures and images for: Utility of TMPRSS4 as a Prognostic Biomarker and Potential Therapeutic Target in Patients with Gastric Cancer
Source: J Gastrointest Surg. 2021 Aug 11;26(2):305–13. doi: 10.1007/s11605-021-05101-2 (PMC8821072; doi:10.1007/s11605-021-05101-2)

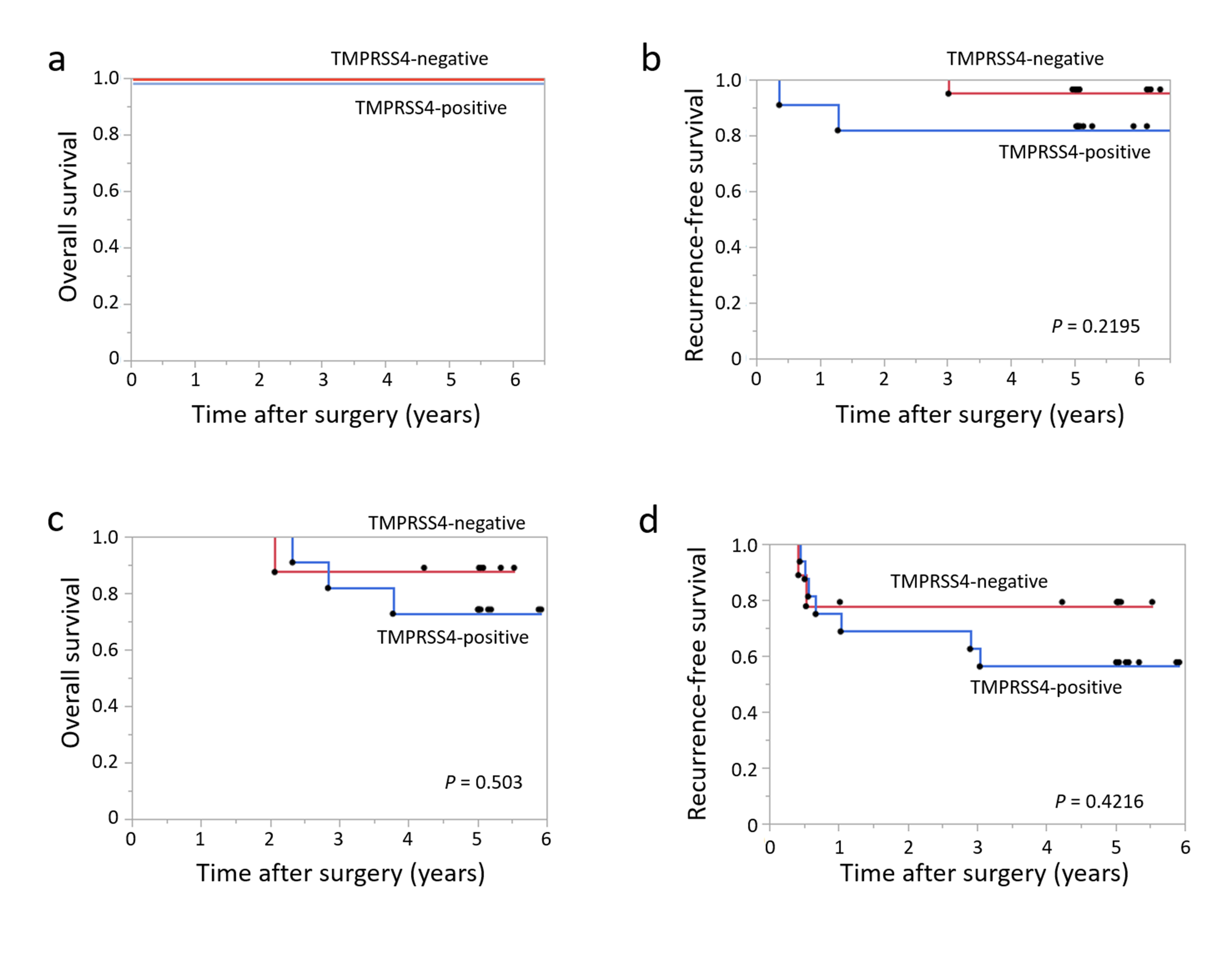

Supplement: Supplementary file 1 — Kaplan-Meier curves for all GC patients classified based on the expression of TMPRSS4 in stages I and II. a. OS curves for all GC patients in stage I. b. RFS curves for all GC patients in stage I. c. OS curves for all GC patients in stage II. d. RFS curves for all GC patients in stage II. The OS and RFS were compared using the Log-rank test. (PNG 540 kb) [file 11605_2021_5101_Fig5_ESM.png]

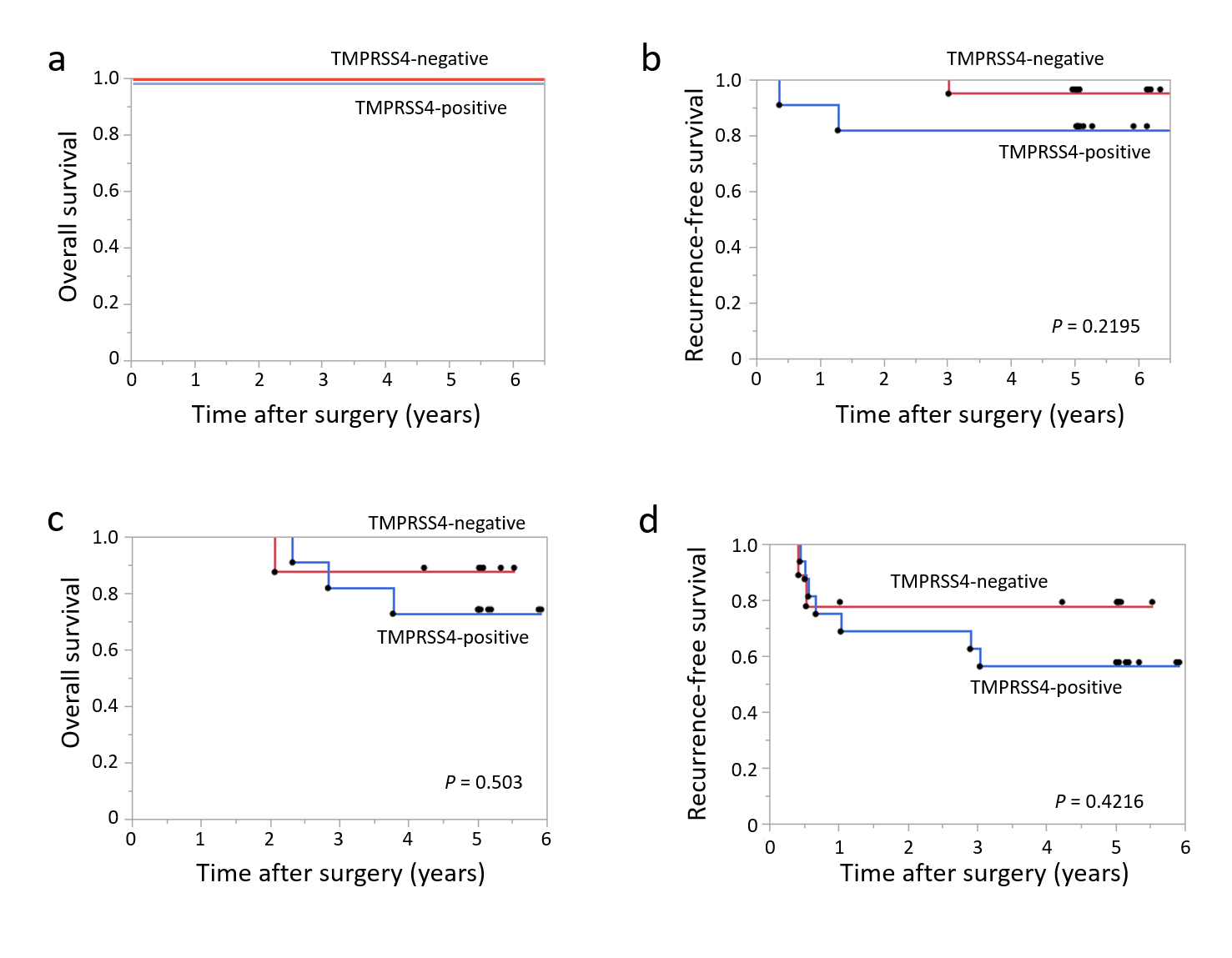

Supplement: Supplementary file 2 — High Resolution image (TIF 253 kb) [file 11605_2021_5101_MOESM1_ESM.tif]

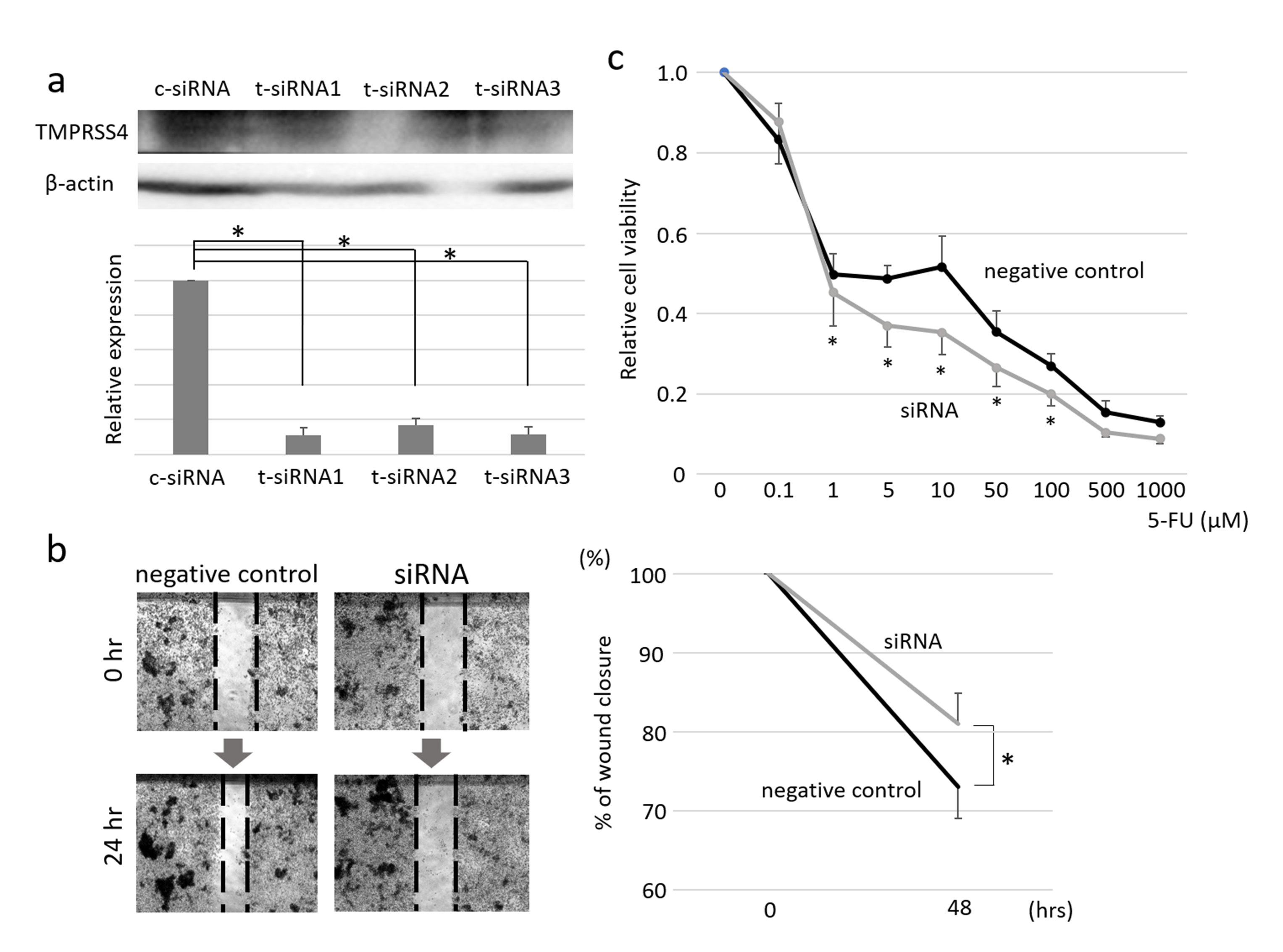

Supplement: Supplementary file 3 — TMPRSS4-silencing reduces the migration and chemosensitivity to 5-FU in GC cells. a. mRNA levels of TMPRSS4 in MKN-45 cells transfected with control siRNA (c-siRNA) or TMPRSS4-targeting siRNA (t-siRNA). Control siRNA transfected cells were used as control. Western blot analysis of TMPRSS4 protein levels in MKN-45 cells transfected with control siRNA (c-siRNA) or TMPRSS4-targeting siRNA (t-siRNA). Control siRNA transfected cells were used as control. Bar graphs indicate the densitometric analysis of western blots of three independent experiments. b. Representative images of migrated cells taken at 48 h after scratching. Bar graphs indicate the mean percentage (± SEM) of wound closure. c. Proliferation assay using CCK-8 kit was performed to examine the inhibitory effect of 5-FU. *p<0.05 (PNG 1216 kb) [file 11605_2021_5101_Fig6_ESM.png]

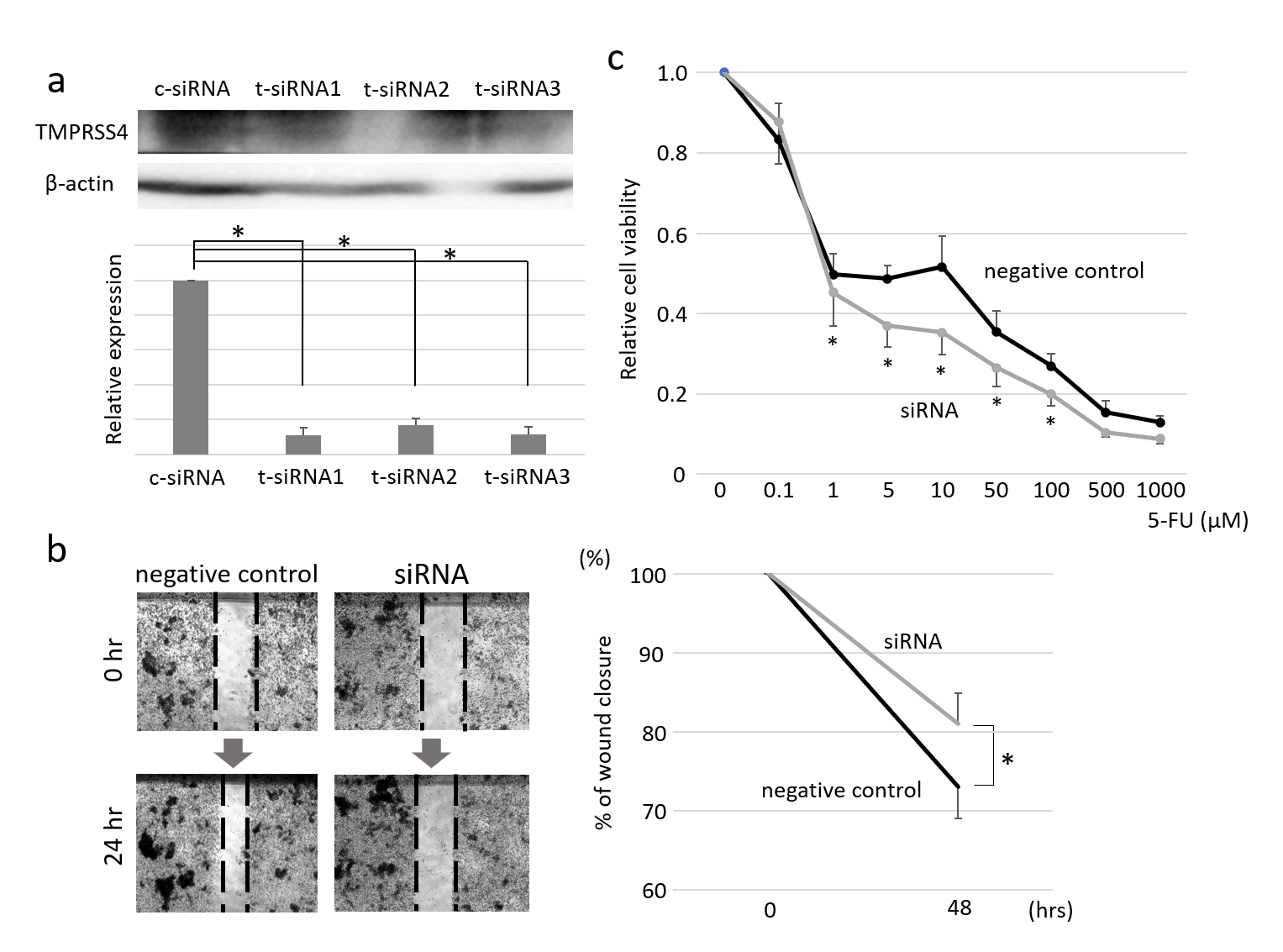

Supplement: Supplementary file 4 — High Resolution image (TIF 528 kb) [file 11605_2021_5101_MOESM2_ESM.tif]
